# Design of an FPGA-Based Fuzzy Feedback Controller for Closed-Loop FES in Knee Joint Model

**DOI:** 10.3390/mi12080968

**Published:** 2021-08-16

**Authors:** Emilia Noorsal, Saharul Arof, Saiful Zaimy Yahaya, Zakaria Hussain, Daniel Kho, Yusnita Mohd Ali

**Affiliations:** 1 School of Electrical Engineering, College of Engineering, Universiti Teknologi MARA, Cawangan Pulau Pinang, Kampus Permatang Pauh, Permatang Pauh 13500, Malaysia; saharul@unikl.edu.my (S.A.); saiful053@uitm.edu.my (S.Z.Y.); zakaria183@uitm.edu.my (Z.H.); yusnita082@uitm.edu.my (Y.M.A.); 2 Electrical, Electronic & Automation Section, Universiti Kuala Lumpur Malaysian Spanish Institute—UniKL MSI, Lot 13-16, KHTP, Kulim 09000, Malaysia; 3 LogikHaus Sdn. Bhd. (1314363-U), c/o Forward School, 2 Lebuh Achech, Georgetown 10450, Malaysia; daniel.kho@logik.haus

**Keywords:** fuzzy logic controller (FLC), functional electrical stimulator (FES), closed-loop FES, FPGA, spinal cord injury (SCI), neuromuscular, knee extension, rehabilitation, hardware description language (HDL), Verilog codes

## Abstract

Functional electrical stimulation (FES) device has been widely used by spinal cord injury (SCI) patients in their rehab exercises to restore motor function to their paralysed muscles. The major challenge of muscle contraction induced by FES is early muscle fatigue due to the open-loop stimulation strategy. To reduce the early muscle fatigue phenomenon, a closed-loop FES system is proposed to track the angle of the limb’s movement and provide an accurate amount of charge according to the desired reference angle. Among the existing feedback controllers, fuzzy logic controller (FLC) has been found to exhibit good control performance in handling complex non-linear systems without developing any complex mathematical model. Recently, there has been considerable interest in the implementation of FLC in hardware embedded systems. Therefore, in this paper, a digital fuzzy feedback controller (FFC) embedded in a field-programmable gate array (FPGA) board was proposed. The digital FFC mainly consists of an analog-to-digital converter (ADC) Data Acquisition and FLC sub-modules. The FFC was designed to monitor and control the progress of knee extension movement by regulating the stimulus pulse width duration to meet the target angle. The knee is expected to extend to a maximum reference angle setting (70°, 40° or 30°) from its normal position of 0° once the stimulus charge is applied to the muscle by the FES device. Initially, the FLC was modelled using MATLAB Simulink. Then, the FLC was hardcoded into digital logic using hardware description language (HDL) Verilog codes. Thereafter, the performance of the digital FLC was tested with a knee extension model using the HDL co-simulation technique in MATLAB Simulink. Finally, for real-time verification, the designed digital FFC was downloaded to the Intel FPGA (DE2-115) board. The digital FFC utilized only 4% of the total FPGA (Cyclone IV E) logic elements (LEs) and required 238 µs to regulate stimulus pulse width data, including 3 µs for the FLC computation. The high processing speed of the digital FFC enables the stimulus pulse width duration to be updated every stimulation cycle. Furthermore, the implemented digital FFC has demonstrated good control performance in accurately controlling the stimulus pulse width duration to reach the desired reference angle with very small overshoot (1.4°) and steady-state error (0.4°). These promising results are very useful for a real-world closed-loop FES application.

## 1. Introduction

In recent decades, functional electrical stimulation (FES) has been widely used in neuromuscular applications to restore the function of paralysed muscles and limbs. The FES systems that are used to restore the loss of neurological control are also known as neuroprostheses. Rehabilitation of spinal cord injury (SCI) patients using FES-assisted devices is one of the most important areas in the health care industry. The FES-assisted device has been used by SCI patients for several rehabilitation exercises, such as grasping, elbow extension, knee extension, standing, walking, cycling, rowing, and sitting down from a standing position. These FES-assisted rehabilitation exercises restore and strengthen the patients’ paralysed muscles, improve motor control and others [1,2]. Besides that, the FES-assisted exercise has also gained interest among clinicians, engineers and researchers. The development of such systems is still on-going, with new innovations and techniques being invented [3,4,5,6,7,8,9,10,11,12,13].

The FES device triggers an action potential in the neural axons by transferring an electrical stimulation charge into the neural tissue. The neurons receive a series of short electrical pulses which are supplied by electrodes to the muscles [14]. The applied stimulus charge artificially depolarizes the nerve membrane and generates an action potential to induce muscle contractions, in particular motor neurons [15,16]. The strength of muscle contraction depends on the intensity of the stimulation. The stimulation intensity is a function of the total charge transferred to the muscle, which depends on the pulse amplitude, duration, and frequency, as well as the shape of the pulse train [17]. These muscle contractions can be adjusted by stimulating one or more muscles that will produce torque at the dedicated limb’s joint.

The major challenge of muscle contraction induced by FES is early muscle fatigue, which greatly limits activities such as FES-assisted standing and walking. Most of the existing FES devices on the market employ an open-loop stimulation strategy to achieve the desired movement for neuromuscular applications [1,10]. Open-loop control systems, which necessitate continuous trial and error of user input, eventually result in early muscle fatigue [7,17,18,19]. The open-loop control FES performance was found unsatisfactory for accurate movement control due to existing parameter variations such as muscle fatigue, inherent time-variance, time-delay, and strong nonlinearities present in the neuromuscular-skeletal system [7,8,20]. This is because the open-loop stimulation technique could not determine the stimulation intensity required by the muscles to meet the desired angle for the limb’s movement [8]. The trial-and-error method employed in the open-loop FES system for stimulus charge settings from user input results in an inaccurate stimulus charge transfer to the muscle. This inaccurate charge transfer might lead to overstimulation and consequently result in an early muscle fatigue phenomenon [7,15,21,22,23]. Therefore, feedback control is necessary to track the limb’s movement and ensure an accurate amount of stimulus charge is delivered to the muscle for the limb’s movement [1,22,24,25].

To solve the problem of the open-loop control system in FES, many researchers have embarked on a closed-loop FES system, where a feedback controller is employed to monitor the stimulated muscle response and provide an accurate amount of charge to the muscle [7,10]. Additionally, closed-loop stimulation with a feedback control unit is very important for reporting the status of the induced muscle, which tends to degrade overtime due to various uncertainties [5,22,26]. The closed-loop FES system with a feedback control unit ensures that only the required amount of stimulus charge is delivered to the muscle, which directly prevents the muscle from early fatigue. Therefore, the real-world FES device is expected to have the capability to regulate the pulse-to-pulse electrical stimulation in real-time to compensate for fatigue, muscle spasm, and retraining effects [1,22,24,25]. Several types of feedback controllers have been commonly used in the closed-loop FES system, which include Proportional-Integral-Derivative (PID) controller [1,27,28,29], Fuzzy Logic [7,8,18,19,28,30,31,32,33,34], Neuro Fuzzy [35], Sliding Mode Controller (SMC) [1,20,25,27,36,37], Gain Scheduling Controller (GSC) [38] and Artificial Neural Network (ANN) controller [36,39] and Adaptive Neuro Fuzzy Inference System (ANFIS) [13].

Comparative analyses conducted by a few researchers indicated that the classical PID controller alone was not a suitable technique for practical FES applications due to its very poor performance in successfully regulating electrically stimulated muscle contractions when non-linearity effects were taken into account [1,28]. The ANN was found to not guarantee stability, too slow for on-line adaptation, need to be trained off-line and when FES is applied to different subjects, neural networks often need to be retrained, which is a time-consuming procedure [20]. The SMC was found to provide fast tuning, and also exhibited the best sinusoidal tracking performance [1]. However, the disadvantages of this SMC are that it suffers from the effect of chattering, and it requires a mathematical model to design the sliding surface [1,20]. The GSC works acceptably well for FES systems, but it is difficult to choose a suitable scheduling variable that captures the nonlinear behaviour of the system. Moreover, GSC involves a lengthy tuning process due to its use of multiple local controllers, and this tuning process may have to be repeated on a daily basis to accommodate variations in the response of the stimulated muscles [1].

Among the existing feedback controllers, the fuzzy logic controller (FLC) has been found to exhibit good control performance as compared to others and has long been known for its ability to handle a complex nonlinear system without developing a complex mathematical model of the system [8,28]. FLC provides solutions for complex, time-varying response engineering systems that would be difficult or impossible to accomplish by a conventional controller [28,40]. It is important to note that a flexible control system is required to adapt to the dynamic and non-linear characteristics of muscles, such as time-varying responses, physical condition, strength and elasticity [8]. Many recent studies have used FLC as the feedback control system for FES-assisted exercises, such as knee extension movement [28,34], cycling movement [8,18,19,30] and elliptical stepping movement [32,41].

Although fuzzy logic has been widely used by researchers in recent decades, particularly for modelling and control in closed-loop FES applications, few research works have been published on the implementation of FLC in embedded systems for real-world closed-loop FES applications. The implementation of FLC as a feedback controller in an embedded system is very important for a wearable closed-loop FES device application [18,42,43]. The possible digital controllers that can be used to develop FLC in embedded systems are microcontroller, Digital Signal Processor (DSP), application-specific integrated circuit (ASIC) and field-programmable gate array (FPGA) technologies [44,45]. However, microcontroller and DSP have some delays in the memory execution due to their sequential nature of processing, which results in low processing speed for complex algorithms [45,46]. Therefore, ASIC and FPGA are preferred due to their excellent performance in terms of parallel computations, concurrency, effective real-time implementation and higher speed [44,47,48,49,50]. Hardware Description Language (HDL) Verilog or VHDL is typically employed to implement digital design in ASICs and FPGAs. Although ASIC could provide a decent low power consumption rate, the customized hardware does not provide flexibility in reprogramming the chip and is not a cost-effective solution due to longer design cycle duration and higher manufacturing costs [44,50]. In contrast, the FPGA provides flexibility in multiple reprogramming according to current design changes, a shorter design cycle duration that enables rapid prototyping and a cost-effective solution for a broad range of applications [44,45,47,48,49]. Therefore, the implementation of FLC in an FPGA device for closed-loop FES applications provides attractive and promising solutions for efficient hardware design, as well as rapid prototyping.

To date, only a few research works have been published on the implementation of FLC in embedded systems for the closed-loop FES application. Some of the published research works by Basith et al. (2017), Basith et al. (2016) and Arrofiqi et al. (2015) have reported the implementation of FLC in an embedded Advanced RISC Machines (ARM) microcontroller for wearable closed-loop FES applications such as knee walking cycles and gait control movements [18,42,43]. FLC implementation in a field-programmable gate array (FPGA) has recently piqued the interest of researchers in both linear and non-linear applications to improve control performance [44,48,49]. Since fuzzy logic does not require a complex mathematical model, it is considered easy to implement for real-world FES applications.

Although the current research work focuses mainly on the feedback controller, other design aspects were also taken into consideration to choose the best digital controller that can fulfil the important design requirements related to FES. Besides the open-loop stimulation technique, the single channel of the existing FES device, which delivers a fixed stimulation pattern at a higher frequency on the same motor unit synchronously, has resulted in the motor unit being overworked and fatigued easily [1,7,13,16,51]. Experimental studies conducted by Downy et al. (2015 and 2017) found that closed-loop multichannel asynchronous interleave stimulation could significantly prolong the functional movement of the lower limb and result in lower rates of fatigue [21,52]. A recent experimental study conducted by Ward et al. (2020) reported that by using a multichannel FES (an array of electrodes), a high level of selectivity in the stimulated muscles could be achieved. Additionally, independent control at each individual electrode enables more muscles to be activated simultaneously, which increases the range of achieved gestures [15]. With real-time feedback and an iterative learning approach, the multichannel closed-loop FES system can help users to achieve precise movements while adapting to their physiology, rather than being limited to a set of fixed stimulation patterns [15]. Therefore, having multiple channels (more than one electrode) enables different motor units to be activated at different times to achieve precise movement, thus improving the efficacy of the FES system [15,21,52].

A recent analytical study on the relationship between flexible stimulation waveform and early muscle fatigue conducted by Noorsal et al. (2020) proved that flexible stimulation waveforms such as ramp up and triangular shapes could reduce the onset of early muscle fatigue compared to rectangular shapes [13]. Hence, a multichannel closed-loop FES that has great flexibility in stimulation waveform generation is essentially required for efficient stimulation and early muscle fatigue reduction in neuromuscular applications [7,15,21,22,23,52].

In summary, based on the previous research findings, having a multichannel closed-loop FES that can demonstrate high flexibility in generating the pulse patterns and stimulation strategy are the important criteria that need to be considered when designing the FES device [15,53]. For multichannel FES, the number of digital stimulators depends on the number of channels (electrodes) employed in the system. This is because each channel requires a dedicated digital stimulator unit, which has a local timer and state machine for precise pulse timing and wave pattern control [15]. Typically, 4 to 8 multichannel electrodes are required to control the movement of each leg and ensure smooth muscle contraction [15,21,53]. Therefore, designing a multichannel closed-loop FES device that can generate flexible waveforms and concurrently process the feedback data demands excellent digital controller performance. After considering the above-mentioned design criteria, an FPGA device is the most suitable and reliable digital controller to handle this complex parallel processing precisely at a higher processing speed.

In this paper, an FPGA-based fuzzy feedback controller (FFC) is proposed for a closed-loop FES system that could monitor the progress of knee extension movement and consequently prevent early muscle fatigue. The knee is expected to extend to the desired reference angle setting from its normal position of 0° once the stimulus charge is applied to the muscle by the FES device. In this research work, the FFC was designed to operate at three reference angles, which include 70°, 40° and 30°. The angle of the knee swing movement was monitored by the FFC by acquiring the feedback sensor data through the analog-to-digital converter (ADC) circuit and processing the acquired data using the FLC’s predefined rulesets. The digital FFC mainly consists of an ADC Data Acquisition and FLC sub-modules. The knowledge of knee swing angles in relation to the stimulus charge (pulse width) was analysed to form a set of rules and consequently control the investigated system. Initially, the FLC was modelled and designed in MATLAB Simulink (Mathworks, Portola Valley, CA, USA) using a non-ideal knee model as a plant system. Thereafter, the FLC was hardcoded into digital logic using hardware description language (HDL) Verilog codes. A testbench was developed to verify the FLC design functionalities. The designed digital FLC was also verified using HDL co-simulation in the MATLAB Simulink environment to compare with the modelled FLC in terms of its rise time, settling time, overshoot and steady-state error. Finally, the digital FFC was designed and downloaded to an Intel FPGA (DE2-115) board (Terasic Inc., Hsinchu, Taiwan) for real-time hardware measurement and verification.

This paper is organized as follows: Section 2 presents the system overview of the closed-loop FES with a feedback controller for the knee extension application. The working principle of knee extension movement in relation to the reference angle and the important data that are acquired from the knee extension movement for the input of the FFC are illustrated and explained. The basic functions and operation of the FLC are also explained in this section. Section 3 elucidates the design methodology and implementation of digital FFC from modelling to hardware measurement setup. Each design method is explained in detail, starting from modelling the FLC in MATLAB Simulink, implementing the FFC in hardware digital logic, system level verification using HDL co-simulation in MATLAB Simulink and a hardware measurement setup for real-time verification. Section 4 discusses simulation and hardware measurement results obtained from MATLAB Simulink, digital simulator and digital oscilloscope (SIGLENT Technologies, Shenzhen, China). In this section, the performance of the designed digital FFC is also discussed and compared with other research work. Finally, Section 5 presents the conclusion of the research work.

## 2. System Overview of Closed-Loop FES for Knee Extension Application

**Figure 1:**
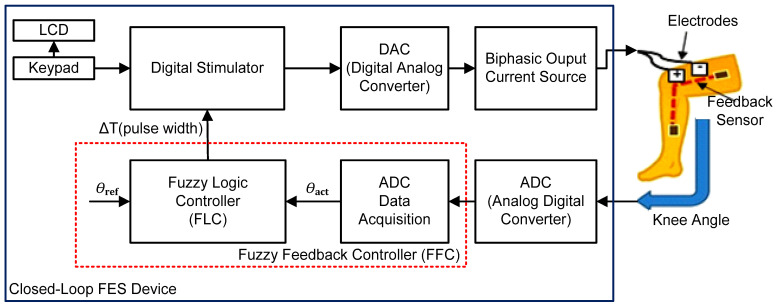
System overview of closed-loop functional electrical stimulation (FES).

The system overview of a typical closed-loop FES-assisted knee extension exercise is illustrated in Figure 1. The closed-loop FES system mainly consists of a Liquid Crystal Display (LCD), Keypad, Digital Stimulator, Digital to Analog Converter (DAC), Biphasic Output Current Source, transcutaneous electrode pads that are attached to the skin, feedback sensors (for example, Goniometer, Rotary Angle, Accelerometer, and Inertial Measurement Unit (IMU) sensors), Analog to Digital Converter (ADC), ADC Data Acquisition and Fuzzy Logic Controller (FLC).

It is important to note that for real-time implementation of FLC in an embedded system, the feedback sensor data need to be acquired from the external ADC circuit. Therefore, the FLC sub-module needs to be integrated with the ADC Data Acquisition sub-module to acquire the digitized feedback data at a specified timing interval. As highlighted in the red dotted-line in Figure 1, the naming convention used for these two sub-modules is Fuzzy Feedback Controller (FFC), which is the main focus of the work in this paper.

The stimulus parameters, which include the amplitude of the current, frequency and wave shapes, are first set on the keypad and displayed on the LCD. The stimulus settings are stored in the Digital Stimulator block and are processed to produce the intended stimulation waveform. The target or maximum reference angle (*θ*_ref_) for the knee extension is set as the FLC input. Based on the selected reference angle (*θ*_ref_) and the actual knee angular position (*θ*_act_), the FLC provides the required amount of pulse width duration (ΔT) to the Digital Stimulator. The amplitude of the digital signal generated by the Digital Stimulator is first converted into an analogue signal by the DAC circuit and then amplified as an output pulse current by the Biphasic Output Current Source circuit. The function of the output current source is to provide a consistent current and also to amplify the stimulus signal before it is sent to the electrode pads. An output current in the range of 0–100 mA is typically used for knee extension exercise to stimulate the quadriceps muscles [43].

In this closed-loop FES system example, a resistive-based angle sensor is used as a feedback sensor to measure the dynamic motion of the SCI patient’s limb during different types of physical activities. Both electrode pads and the resistive-based angle sensor are attached to the human skin. The generated stimulus charge is transferred to the human muscle via the electrode pads for stimulation purposes. The electrode pads enable the flow of current through the muscle for contraction purposes. The feedback sensor keeps track of the limb’s movement by changes in the resistance value of its resistive element. The output voltage of the sensor is then converted into digital format by the ADC circuit before being sampled by the ADC Data Acquisition sub-module. The sampled feedback data (in digital format) from the ADC Data Acquisition sub-module are then processed by the FLC to accurately monitor and control the FES-induced movement of the limb.

Essentially, the FFC monitors the limb’s movement by tracking the actual angular position (*θ*_act_) generated by the feedback sensor and comparing it with the target reference angle (*θ*_ref_). The FFC controls the required amount of charge to the Digital Stimulator by modulating the stimulus pulse width duration (ΔT) to ensure that the target reference angle (*θ*_ref_) is met.

### 2.1. Knee Extension

**Figure 2:**
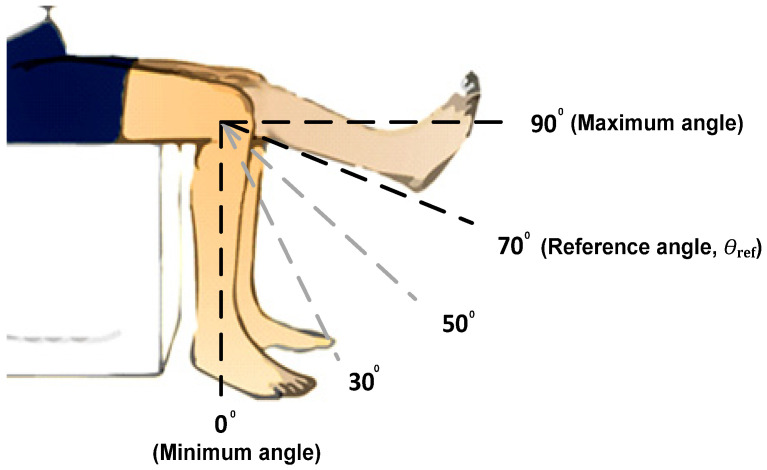
Example of knee extension with a reference angle of 70°.

Figure 2 illustrates several possible knee angle movements that will be produced due to muscle contractions when stimulus charges are applied to the quadriceps muscle.

The angle in Figure 2 shows the estimated knee movement of an SCI patient using the closed-loop FES device. In rest mode, the knee angle is at 0° and remains at this angle until a stimulus charge is applied to the quadriceps muscles by the FES device. The injected stimulus charge will contract the muscle, which consequently results in knee extension movement. The actual angle (*θ*_act_) produced by the limb’s movement will be used as a feedback input to the FLC to determine the required amount of charge that needs to be adjusted during the stimulation operation. As illustrated in Figure 2, the example of the target angle (*θ*_ref_) is set at 70°. The difference between *θ*_ref_ and *θ*_act_ is known as an Error (E), while the difference between current and previous errors is known as a Change in Error (dE). The Error (E) and Change in Error (dE) can be defined by the following equations:

(1) $$\mathrm{Error},E=\mathrm{Reference}\;\mathrm{Angle}\;\left(\theta_{\mathrm{ref}}\right)-\mathrm{Actual}\;\mathrm{Angle}\;\left(\theta_{\mathrm{act}}\right)$$

(2) $$\mathrm{Change}\;\mathrm{in}\;\mathrm{Error},\mathrm{dE}=\mathrm{Current}\;\mathrm{Error}\;\left(E_{k}\right)-\mathrm{Previous}\;\mathrm{Error}\left(E_{k-1}\right)$$

The FLC processes these input data (E and dE) to determine the amount of charge (pulse width duration) required to meet the *θ*_ref_. Positive E indicates that the amount of applied charge is still insufficient, while negative E indicates an overstimulation of the charge. Therefore, the stimulus pulse width (ΔT) will be adjusted accordingly (increasing or decreasing) by the FLC until the target angle is met.

### 2.2. Fuzzy Logic Controller

**Figure 3:**
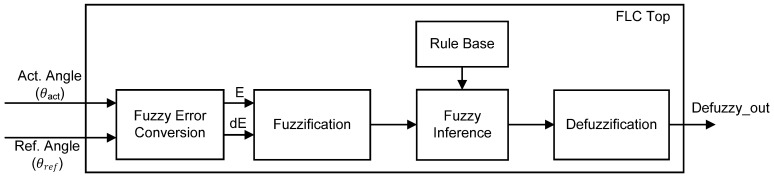
System overview of fuzzy logic controller (FLC).

Control of knee extension for the closed-loop FES system as proposed in this work was implemented using FLC. The FLC mainly consists of Fuzzy Error Conversion, Fuzzification, Rule base, Fuzzy Inference and Defuzzification sub-modules as depicted in Figure 3. The Fuzzy Error Conversion sub-module first converts the acquired actual angle (*θ*_act_) of knee position into Error (E) and Change in Error (dE). Both Error (E) and Change in Error (dE) have a range of minimum and maximum values based on how the system responds to these input parameters. For the digital implementation, these input parameters are further scaled into 8-bit data ranging from 0 to 255.

The Fuzzification sub-module transforms the crisp input values (Error (E) and Change in Error (dE)) into membership grades in the range of 0 to 1 through the use of membership functions. The membership function distributes the inputs within the scaled value range via several fuzzy sets. Ideally, 3, 5 or 7 fuzzy sets are sufficient with shapes such as triangular, trapezoidal or gaussian. The Rule-Base sub-module stores knowledge in the form of a set of rules that represent the actions of the FLC according to the inputs. Defining fuzzy rules is basically done through the IF-THEN rule together with “AND” or “OR” connections where all control possibilities can be constructed in the fuzzy rule matrix. The Fuzzy Inference performs an input-to-output mapping and generates a control decision based on the parameters described in the membership function, fuzzy set, and fuzzy rule. The two most commonly used fuzzy inference methods are Mamdani and Takagi–Sugeno. For hardware implementation, the Takagi–Sugeno model is preferred over the Mamdani model due to its simple method of calculation, which leads to fast and efficient computation. Additionally, the Takagi–Sugeno model provides a good trade-off between hardware simplicity and control efficiency [54]. The output of Fuzzy Inference requires a defuzzification process to transform the fuzzified values into a single crisp output value. The Defuzzification sub-module usually employs the Centre of Gravity (CoG) defuzzification method due to its smooth output and ease of calculation.

## 3. Materials and Methods

This section discusses the design methodology and implementation of the digital FFC using the FPGA (DE2-115) board. The FFC was designed to operate at three reference angles: 70°, 40° and 30°. In this research work, there are four main design phases that need to be executed for the FPGA-based digital FFC implementation. The design process started with modelling the FLC in MATLAB Simulink for a closed-loop FES, knee extension application. Once the optimized FLC was obtained in the modelling phase, the FLC was designed and developed using a hardware description language (HDL) for digital hardware logic implementation. The designed digital FLC was then imported into MATLAB Simulink for system level verification using the HDL co-simulation method. The performance of the designed digital FLC was compared with the modelled FLC. Finally, once the performance of the digital FLC was satisfied, the digital FFC (integration of FLC and ADC Data Acquisition sub-modules) was designed and downloaded onto the FPGA (DE2-115) boardfor real-time hardware measurement. At each design phase, the functionality and performance of the designed FLC were verified and evaluated. The following sub-sections will provide detailed explanations of each design phase.

### 3.1. Design and Modelling of Fuzzy Logic Controller for Knee Extension

In this research work, the knee extension against gravity model was chosen as a plant for this closed-loop FES system because of its fundamental role in other motor activities, which include standing up, sitting down, walking, standing posture, climbing stairs, etc. [55]. The knee extension model was developed using the Ferrarin and Pedotti (2000) method, in which a non-linear second-order system to model the dynamics of the knee and lower leg, and a one-pole transfer function to model the relationship between stimulation pulse width and quadriceps torque were employed [55]. This method employs a mathematical model of the lower limb to describe the dynamic equilibrium of the moments acting on the knee joint, taking into account the gravitational and inertial characteristics of the anatomical segments together with the damping and stiffness properties of the knee [1,55].

**Figure 4:**
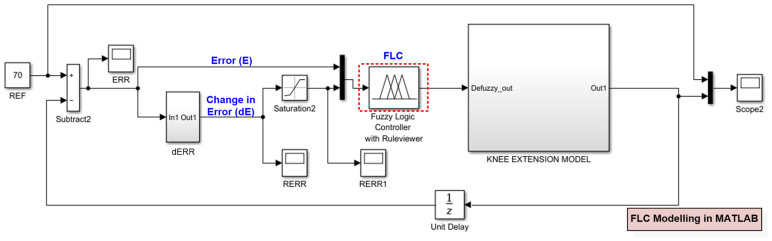
FLC modelling using the knee extension model as a plant in MATLAB Simulink.

Initially, the FLC was developed and modelled in MATLAB Simulink for the closed-loop FES knee extension application as depicted in Figure 4. The internal blocks of FLC, such as Fuzzification, Rule-base and Defuzzification, were developed using the Fuzzy Logic Toolbox to define the membership function and to formulate fuzzy rules. The FLC was designed to operate at three reference angles (*θ*_ref_), which are 70°, 40° and 30°. The defuzzy output from the FLC was directly connected to the knee extension model to generate the desired torque output. The knee trajectory output response or actual angle (*θ*_act_) was fed back to the input of FLC to ensure the desired target angle was met.

The actual angle was then converted into Error (E) and Change in Error (dE). The Error (E) and Change in Error (dE) were connected to the input of FLC. In this research work, the FLC structure was developed using the Sugeno style, in which the output membership functions (MFs) of the fuzzy are singletons. The Sugeno method was chosen due to its simplicity, compactness, being computationally more efficient and easier to implement on digital hardware [54,56]. Details of each FLC design block implementation are explained in the following sub-sections.

#### 3.1.1. Fuzzification

**Figure 5:**
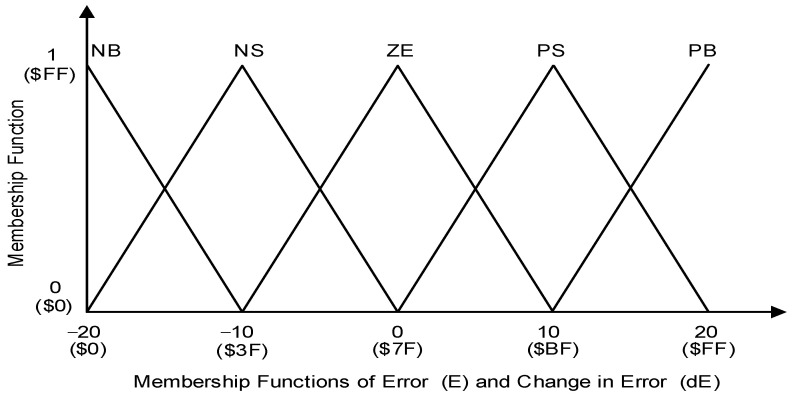
Membership Function (MF) of Error (E) and Change in Error (dE) with digital scaling format. The input MFs are Negative Big (NB), Negative Small (NS), Zero (ZE), Positive Small (PS) and Positive Big (PB).

In this project, triangular MFs were used for the two inputs of Error (E) and Change in Error (dE). Symmetrical triangular shapes were used throughout this application because of their simple calculation, linear characteristics, proper intersection between each fuzzy set, small steady-state error and efficiency of implementation in Verilog HDL [8]. Figure 5 illustrates how triangular input membership functions were formed for Error (E) and Change in Error (dE) in the fuzzification process using a trial-and-error method. For each input of Error (E) and Change in Error (dE), five triangular MFs were used, which included Positive Big (PB), Positive Small (PS), Zero (ZE), Negative Small (NS) and Negative Big (NB). In this work, both Error (E) and Change in Error (dE) were designed to have similar MFs in the range of −20 to 20. The detailed ranges of values for the above-mentioned MFs are listed in Table 1. The values of the x and y axes for all MFs were scaled into 8-bit digital values from 0 ($00) to 255 ($FF) for digital implementation in the FPGA, as depicted in Figure 5 and listed in Table 1 (“$” indicates hexadecimal value).

The MF ranges from the modelled FLC were converted into 8-bit digital MF ranges by using Equation (3). Examples of MF conversion for values of −20, 0 and 20 into 8-bit digital MF scaling are shown in Equations (4)–(6). The “$” sign indicates hexadecimal number representation.

(3) $$\mathrm{Digital}\;\mathrm{MF}\;\mathrm{scale}\;\left(x\right)=\frac{x+\;\mathrm{Max}\;\mathrm{MF}\;}{\mathrm{Total}\;\mathrm{MF}\;\mathrm{range}}\times 255$$

(4) $$\mathrm{Digital}\;\mathrm{MF}\;\mathrm{scale}\;\left(-20\right)=\frac{-20+20}{40}\times 255=0\;\left[\$00\right]$$

(5) $$\mathrm{Digital}\;\mathrm{MF}\;\mathrm{scale}\;\left(0\right)=\frac{0+20}{40}\times 255=127\;\left[\$7F\right]$$

(6) $$\mathrm{Digital}\;\mathrm{MF}\;\mathrm{scale}\;\left(20\right)=\frac{20+20}{40}\times 255=255\;\left[\$\mathrm{FF}\right]$$

#### 3.1.2. Fuzzy Inference (Rule Base)

**Figure 6:**
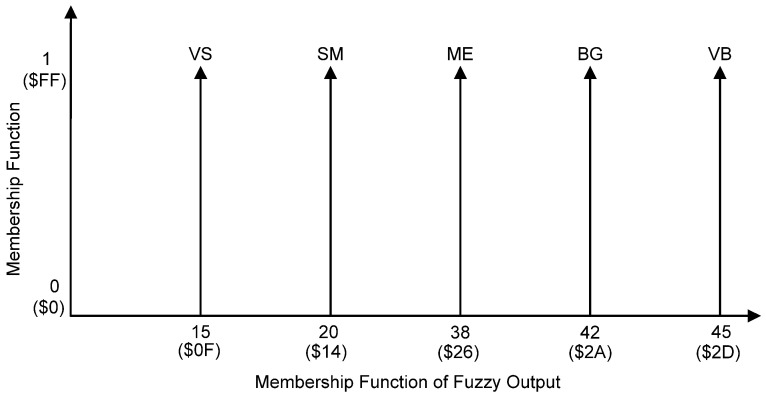
MF of fuzzy output (reference angle = 70°) with digital scaling format. The singleton output MFs are Very Small (VS), Small (SM), Medium (ME), Big (BG) and Very Big (VB).

For the fuzzy inference mechanism, fuzzy rules (Rule Base) were first created to decide the action that needed to be taken in response to a given set of degrees of MF. The number of rules was determined by the number of MFs for each input. The determination of the rules was done by understanding the control process and knowledge of the relationship between knee trajectory response and the applied stimulus charge. The output MFs in this Takagi–Sugeno model are fuzzy singletons that take on an MF value of 1 at a single location and 0 at all other locations. The Sugeno singleton method was employed due to its simplicity and reduction in computational complexity [54,56]. Each output MF was treated as a singleton located at the centre of the MF as depicted in Figure 6 (reference angle set at 70°). The linguistic terms for five-singleton output MFs are Very Small (VS), Small (SM), Medium (ME), Big (BG) and Very Big (VB).

Since the FLC has two inputs (Error (E) and Change in Error (dE)) with five membership functions (NB, NS, ZE, PS, PB) for each input, 25 rules (5 × 5 = 25) were produced as tabulated in Table 2. The 25 singleton fuzzy outputs are also known as c1–c25. Any combination of two linguistic variables fires at least one rule.

Based on the set of rules from Table 2, examples of fuzzy rules that take the form of IF-THEN statements for min1–min5 using the Takagi–Sugeno method are shown in Equations (7)–(11). The same IF-THEN statements are also applied to min6–min25 to produce singleton outputs for c6–c25.

(7) min1= If (input1 is NB) AND (input2 is NB) then (output c1 is VS)

(8) min2= If (input1 is NS) AND (input2 is NB) then (output c2 is VS)

(9) min3= If (input1 is ZE) AND (input2 is NB) then (output c3 is VS)

(10) min4= If (input1 is PS) AND (input2 is NB) then (output c4 is SM)

(11) min5= If (input1 is PB) AND (input2 is NB) then (output c5 is ME)

#### 3.1.3. Defuzzification

After the MF output grade of each rule had been determined, the next step was to combine all the output grades into a single value that would be used as a pulse width signal to control the amount of charge applied to the muscle. In this work, since the closed-loop FES was designed to operate at three reference angles (70°, 40° and 30°), three sets of MF output singleton positions were optimized to meet the reference angle settings as listed in Table 3.

Finally, the defuzzy output was calculated using the centre of gravity (COG) method. The COG method has the advantage of reducing computational complexity and producing a fast output [56]. This COG method was implemented by multiplying the fuzzy output from the rules’ evaluation with its corresponding singleton value, then dividing the sum of this value by the sum of all fuzzy outputs from the rules’ evaluation, as shown in Equation (12).

(12) $$\mathrm{Crisp}\;\mathrm{Output}=\frac{\sum i\;\left(\mathrm{fuzzy}\;\mathrm{output}\right)\times \left(\mathrm{Sin}\mathrm{gleton}\;\mathrm{position}\;\mathrm{on}\;x\;\mathrm{axis}\right)}{\sum i\;\left(\mathrm{fuzzy}\;\mathrm{output}\right)}$$

### 3.2. Design of the Digital Fuzzy Feedback Controller

**Figure 7:**
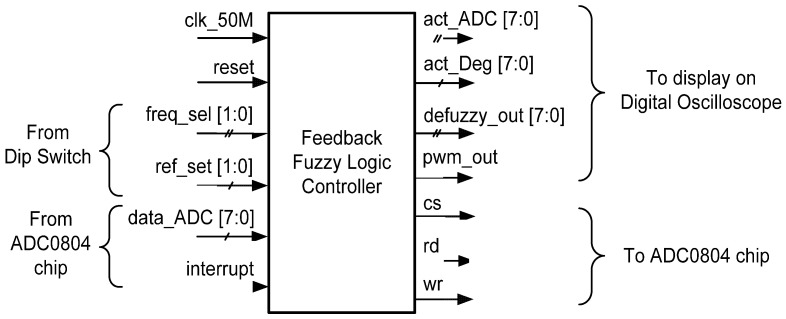
Top level of digital Fuzzy Feedback Controller (FFC).

The digital FFC was designed using hardware description language (HDL) Verilog codes and implemented on the Intel’s FPGA (DE2-115) board. Figure 7 depicts the FFC top level.

As mentioned in Section 2 and illustrated in Figure 1, the FFC mainly consists of ADC Data Acquisition and FLC. The main function of this digital FFC is to acquire the knee trajectory angle from the feedback path (feedback sensor and ADC circuit), to track the knee angle movement and to produce a relevant pulse width signal to the Digital Stimulator block for charge stimulation control in order to meet the target reference angle (70°, 40° or 30°). For hardware measurement setup, the FFC was designed to be interfaced with dip switches and an ADC (ADC0804) circuit (Texas Instruments, Dallas, TX, USA). The ADC (ADC0804) chip was used to acquire and convert the analogue voltage of the feedback sensor into a digital 8-bit data format. Dip switches were used to set the reference angle (“ref_set [1:0]”) and the stimulation frequency of the pulse width (“freq_sel [1:0]”). Therefore, the user can select the desired target angle (70°, 40° or 30°) and stimulation frequency (20 Hz, 30 Hz, 40 Hz or 50 Hz) by setting the dip switches accordingly.

Some of the FFC signals, such as the acquired ADC data (“act_ADC [7:0]”), the converted ADC angle data into degrees (“act_Deg [7:0]”), the defuzzy output (“defuzzy_out [7:0]”) and the pulse width signal (“pwm_out”), were set as output to observe the designed FFC functionality on a digital oscilloscope.

**Figure 8:**
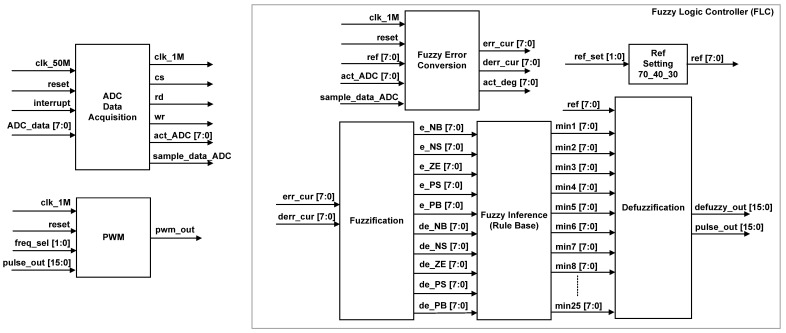
Internal architecture of the digital FFC.

The internal architecture of the digital FFC as depicted in Figure 8 consists of two main sub-modules, which include ADC Data Acquisition and FLC. The internal sub-modules of FLC are Fuzzy Error Conversion, Reference Setting, Fuzzification, Fuzzy Inference (Rule Base) and Defuzzification (grouped together in a grey box). The Pulse Width Modulator (PWM) sub-module was added to emulate the generation of the pulse width signal by the Digital Stimulator block in Figure 1. The ADC Data Acquisition sub-module is the main control unit for the FFC that triggers the data acquisition process from the ADC (ADC0804) circuit every 100 ms.

In this design, the clock frequency of 50 MHz from the FPGA (DE2-115) board was used as a master clock input. The master clock at 50 MHz (“clk_50M”) was down-converted into a 1 MHz clock (“clk_1M”) for the usage of ADC Data Acquisition, Fuzzy Error Conversion and PWM sub-modules. After the system is powered on and reset, the digital FFC begins tracking knee angle movement by acquiring the actual knee angle data from the ADC circuit every 100 ms. Right after the ADC data are acquired, the FLC sub-module is triggered to process the acquired feedback data and produce the required pulse width data (“pulse_out [15:0]”) for the PWM sub-module. The stimulus pulses are then delivered at frequencies ranging from 20–50 Hz. The following sub-sections explain the operation of the FFC, beginning with ADC data acquisition and progressing to fuzzy error conversion, fuzzification, rule base inference, defuzzification, and finally pulse width signal generation.

#### 3.2.1. ADC Data Acquisition

**Figure 9:**
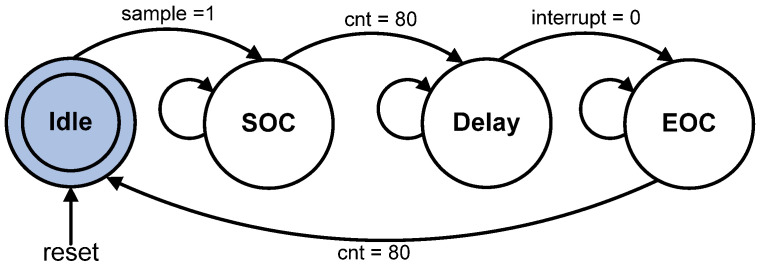
Analog-to-digital converter (ADC) Data Acquisition state diagram.

The digitized sensor data (“ADC_data [7:0]”) from the ADC circuit are acquired by the ADC Data Acquisition sub-module every 100 ms using the interface control signals (“cs”, “rd”, “wr” and “interrupt”) to communicate with the ADC (ADC0804) chip. Figure 9 illustrates the state diagram of the finite state machine (FSM) for the ADC data acquisition process.

In this design, only four states are required to acquire the digitized 8-bit data from the ADC (ADC0804) chip. After the system is powered on and reset, the FSM enters the Idle state and remains there until the internal “sample” signal is set to logic HIGH every 100 ms. Then, the FSM enters the state of SOC and remains in the state for 80 µs. An internal counter (“cnt”) is used as a reference timer by the FSM to set the duration for each state. During the SOC state, the control signals “cs” and “wr” are set to logic LOW to activate and inform the ADC (ADC0804) chip to start the conversion process. Once the internal counter value (“cnt”) is equal to 80, the FSM moves to state Delay and remains in this state until the “interrupt” signal from the ADC (ADC0804) chip is logic LOW. The logic LOW of the “interrupt” signal indicates that the conversion process from analogue to digital value from the ADC (ADC0804) chip has been completed and the digitized data are ready to be sampled. When the “interrupt” signal is logic LOW, the FSM enters the EOC state and remains in this state for 80 µs. During the EOC state, the ADC data are sampled and acquired by activating control signals “cs” and “rd” to logic LOW. Once the internal counter value (“cnt”) is equal to 80 the FSM moves to state Idle and activates the “sample_data_ADC” signal to store the acquired ADC data and for the Fuzzy Error Conversion sub-module to undertake the operation. Table 4 tabulates the details of state movements with the respective output control signals for each state.

#### 3.2.2. Fuzzy Error Conversion

The function of the Fuzzy Error Conversion sub-module is to convert the crisp input data (from the ADC) into a fuzzification data format (Error (E) and Change in Error (dE)). The sampled ADC data (“act_ADC [7:0]”) in the range of 0–255 are first converted to an actual angle (“act_deg [7:0]”) in degrees (0°–90°). The mathematical operation for the conversion process is shown in Equation (13).

(13) act_deg=(act_ADC ×90°)/255

**Figure 10:**
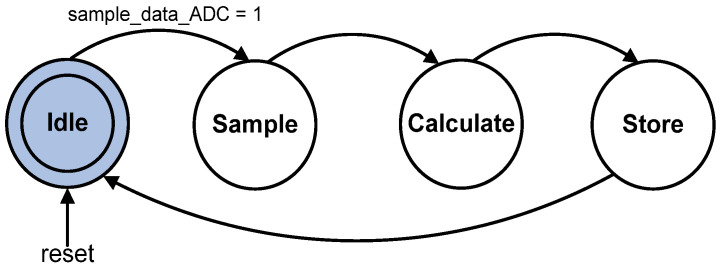
Fuzzy Error Conversion state diagram.

Figure 10 illustrates the FSM state diagram for the sampling and conversion process. Once the “sample_data_ADC” is activated, the FSM moves from state Idle to state Sample. In the Sample state, the reference and the actual degree angle data (“ref [7:0]” and “act_deg [7:0]”) are stored in internal registers. The FSM moves to state Calculate on the next clock cycle, to calculate the Error (“err_cur [7:0]”) by subtracting the actual angle (“act_deg [7:0]”) from the reference angle (“ref [7:0]”). The FSM then goes to state Store to compute the Change in Error (“derr_cur [7:0]”) and saves the current existing Error (“err_cur [7:0]”) into another internal register known as Previous Error.

It is important to note that, besides converting the acquired ADC data into Error (“err_cur [7:0]”) and Change in Error (“derr_cur [7:0]”) using the FSM, the Fuzzy Error Conversion sub-module indirectly controls and determines the duration of the FLC sub-module mathematical operation as a whole. This is because the other sub-modules, such as Reference Setting, Fuzzification, Fuzzy Inference (Rule Base) and Defuzzification, consist of combinational logic gates, which directly execute the mathematical operations in parallel once the input data are made available. Therefore, in this FLC design, the FSM of the Fuzzy Error Conversion sub-module requires only three clock cycles (from state Sample to state Store) to produce the defuzzy and pulse width output values as depicted in Figure 10.

#### 3.2.3. Fuzzification

The Fuzzification sub-module converts the crisp input values into the membership values of the fuzzy sets. Both Error (“err_cur [7:0]”) and Change in Error (“derr_cur [7:0]”) generated by the Fuzzy Error Conversion sub-module are fed to the Fuzzification sub-module for the fuzzification process. The crisp input values for Error and Change in Error were first scaled to be in the range of −20 to 20 before being converted into a digital scaling format of 0 ($00) to 255 ($FF) as listed in Table 1 (Section 3.1.1). Five triangular MFs were employed for both Error (E) and Change in Error (dE), which include PB (Positive Big), PS (Positive Small), ZE (zero), NS (negative Small) and NB (Negative Big).

**Figure 11:**
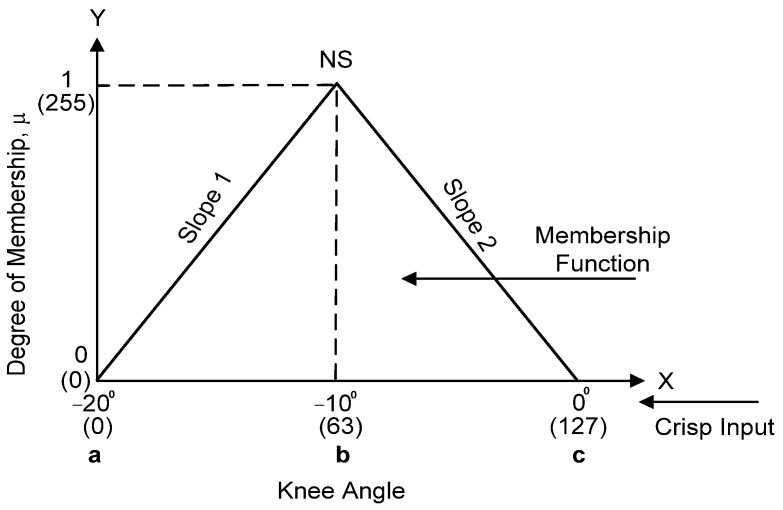
Example of Negative Small (NS) triangular MF for crisp input data.

Figure 11 illustrates an example of how the crisp input data are fuzzified into a triangular MF in the fuzzification process.

Equation (14) defines the degree of triangular MF at five different ranges of crisp input (X) values. Equation (15) shows an example of the triangular MF that has been scaled and converted into a digital format as illustrated in Figure 11.

(14) $$\mathrm{Triangular}\;\left(X;a;b;c\right)=\left\{ \begin{array}{cc} 0, & X\leq a\\ \frac{X-a}{b-a}\times \left(X-a\right), & a<X<b\\ 255, & X=b\\ \frac{c-X}{c-b}\times \left(c-X\right), & b<X<c\\ 0, & X\geq c \end{array}\right.$$

(15) $$\mathrm{Triangular}\;\left(X;0;63;127\right)=\left\{ \begin{array}{cc} 0, & X\leq 0\\ \frac{255-0}{63-0}\times \left(X-0\right), & 0<X<63\\ 255, & X=63\\ \frac{255-0}{127-63}\times \left(127-X\right), & 63<X<127\\ 0, & X\geq 127 \end{array}\right.$$

Therefore, the fuzzified output of Error (“e_NB”, “e_NS”, “e_ZE”, “e_PS”, “e_PB”) and Change in Error (“de_NB”, “de_NS”, “de_ZE”, “de_PS”, “de_PB”), represent the membership degree ranging from 0 (0) to 1 (255). These fuzzified outputs are connected to the Fuzzy Inference (Rule-Base) sub-module as input signals.

The example of a pseudocode for scaling of input Error and fuzzification processes is shown in Algorithm 1. The pseudocode starts with the scaling of input Error (−20 to 20), digital format scaling (0–255) and finally determining the membership degree of NS fuzzification.
**Algorithm 1.** Scaling of Input Error and Fuzzification (Negative Small (NS) MF)1.#Scale Input Error to −20 and 20.2.if (err_cur < −20)3.    err_cur1 = −20;4.else if (err_cur > 20)5.    err_cur1 = 20;6.else7.    err_cur1 = err_cur;8.#Convert Input Error to Digital Scale Format (0–255). Refer Equations (3)–(6)9.err_scl = (((err_cur1 + 20) * 255)/40);10.#Declare NS range of error11.a**=**8’d0**;** b**=**8’d63**;** c**=**8’d127;12.#Calculate the Error MF for NS. Refer Equations (14) and (15)13.if ((err_scl <= a) || (err_scl >= c))14.    e_NS = 0;15.else if (err_scl > a && err_scl < b)16.    e_NS = (255/(b-0) * (err_scl - a));17.else if (err_scl > b2 && err_scl < c)18.    e_NS = (255/(c-b) * (c - err_scl));19.else20.    e_NS = 255;

#### 3.2.4. Fuzzy Inference (Rule Base)

In the Fuzzy Inference sub-module, the IF-THEN rule methods are applied to the fuzzified input of Error (“e_NB”, “e_NS”, “e_ZE”, “e_PS”, “e_PB”) and Change in Error (“de_NB”, “de_NS”, “de_ZE”, “de_PS”, “de_PB”) as explained in Section 3.1.2 and illustrated in Table 2. Since both input grades (Error and Change in Error) are connected by a logical “AND” operator from the IF-THEN rule statement (refer to Equations (7)–(11)), the minimum function between two antecedents is used to obtain the result of the evaluated rule [57,58]. The input Errors (“e_NB”, “e_NS”, “e_ZE”, “e_PS”, “e_PB”) are compared with the Change in Errors (“de_NB”, “de_NS”, “de_ZE”,”de_PS”, “de_PB”) to get the minimum outputs for “min1”–“min25”. The minimum outputs of the Fuzzy Inference sub-module (“min1”–“min25”) are connected to the Defuzzification sub-module.

The example of a pseudocode for the Fuzzy Inference (Rule-base) operation for rules “min1”–“min3” is shown in Algorithm 2.
**Algorithm 2.** Fuzzy Inference (Rule Base)1.#min1 rule check. Refer Equation (7).2.if (e_NB <= de_NB)3.    min1 = e_NB;4.else5.    min1 = de_NB;6.#min2 rule check. Refer Equation (8).7.if (e_NS <= de_NB)8.    min2 = e_NS;9.else10.    min2 = de_NB;11.#min3 rule check. Refer Equation (9).12.if (e_ZE <= de_NB)13.    min3 = e_ZE;14.else15.    min3 = de_NB;

#### 3.2.5. Defuzzification

The Defuzzification sub-module combines all 25 output grades (${“\min}_{1}-{\;\min}_{25}”$) obtained from the Fuzzy Inference sub-module into a single value as shown in Equation (12) (Section 3.1.3) to produce the final defuzzy output value. The Defuzzification sub-module is designed according to Equation (12), which takes the weighted average of all fuzzy outputs (${“\min}_{1}-{\;\min}_{25}”$) from the Fuzzy Inference sub-module. Equations (16) and (17) redefine Equation (12) using Fuzzy Inference output grades (${“\min}_{1}-{\;\min}_{25}”$) and MF singleton outputs (${“c}_{1}-c_{25}”)$:

(16) $$\mathrm{defuzzy}\;\mathrm{output}=\frac{\sum \min_{i}\times c_{i}\;}{\sum {\;\min}_{i}}\;,\;\left(i:1-25\right)\;$$

(17) $$\mathrm{defuzzy}\;\mathrm{output}=\frac{\left(\min_{1}\times c_{1})+(\min_{2}\times c_{2})+\ldots (\min_{25}\times c_{25}\right)}{\min_{1}+\min_{2}+\min_{3}+\ldots {\;\min}_{25}}\;,\;\left(i:1-25\right)$$

From Equation (16), each individual Fuzzy Inference output ($\min_{i}$) is multiplied with the corresponding MF singleton output ($c_{i}$). Then the sum of these products is divided by the sum of all the Fuzzy Inference outputs $\left({“\min}_{1}-{\;\min}_{25}”\right)$ for the final defuzzy output results. Equation (17) describes the weighted average calculation for the 25 minimum outputs (${“\min}_{1}-{\;\min}_{25}”$) with MF singleton outputs $\left({“c}_{1}-{\;c}_{25}”\right)$ in greater detail. The settings of MF singleton outputs $\left({“c}_{1}-{\;c}_{25}”\right)$ can be referred to in Table 2 (Section 3.1.2).

Since three reference angles are used for this closed-loop knee extension exercise, three sets of MF singleton outputs have been obtained during the FLC modelling as mentioned and listed in Table 3 (Section 3.1.3). Therefore, the Defuzzification sub-module needs to cater to three reference angles (70°, 40° and 30°) when selected by the user. The external reference setting (“ref_set [1:0]”) needs to be decoded by the Ref. to Singleton Decoder sub-module to produce relevant MF singleton output settings as listed in Table 3 (Section 3.1.3). Then, the 25 singleton outputs (“$c_{1}-{\;c}_{25}$”) are assigned according to their dedicated rule base as mapped in Table 2 (Section 3.1.2) by the Defuzzification sub-module. Referring to Equation (17), for the numerator part, multiplier and adder logic gates are used to realize the multiplication and addition (MAC) operation. For the denominator part, adders are used to sum up all the minimum outputs ($\min_{1}-{\;\min}_{25}$) from the Fuzzy Inference sub-module. The defuzzy output (“defuzzy_out [15:0]”) is then multiplied by 10 to generate a pulse output (“pulse [15:0]”) data for the actual pulse width duration. This pulse output data are connected to the PWM sub-module for pulse width generation.

#### 3.2.6. Pulse Width Modulator (PWM)

As mentioned earlier, the PWM sub-module was added to this design to emulate the generation of pulse width signals by the Digital Stimulator block. The “defuzzy output” in the range of 10 to 50 from the Defuzzification sub-module, is multiplied by 10 to get the actual stimulus pulse delay (“pulse [15:0]”) ranging from 100–500 µs. The stimulus pulse duration ranging from 100 to 500 µs has been used by many researchers for neuromuscular FES applications [1,13,25,51,59]. The frequency selection signal (“freq_sel [1:0]”) is used to set the stimulus frequencies in the range of 20–50 Hz. The frequency setting of PWM depends on the user’s selection of the external dip switches.

### 3.3. System Level HDL Co-Simulation Development for Fuzzy Feedback Controller

**Figure 12:**
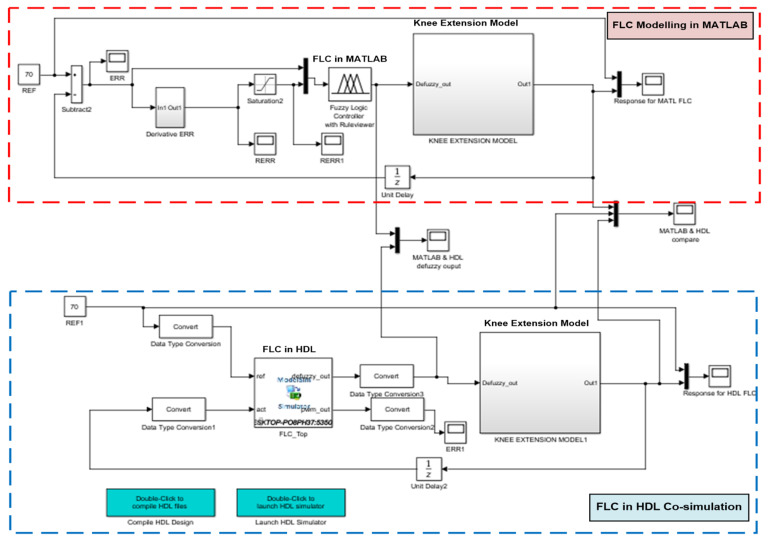
System level hardware description language (HDL) co-simulation development of FLC using the knee extension model as a plant in MATLAB Simulink.

For system level verification and performance analysis, the HDL codes were imported into MATLAB Simulink. Figure 12 depicts the system level setting of closed-loop FES for knee extension application. Similar knee extension model and modelled FLC with three reference settings (70°, 40° and 30°) were used for the MATLAB Simulink as mentioned in Section 3.1, except for the new HDL FLC block that was included in this system level verification. This HDL system level verification in MATLAB Simulink is also known as HDL co-simulation. This HDL co-simulation was used to compare the performance of the digital FLC with the original FLC modelled on MATLAB Simulink. The output of the digital FLC (“defuzzy_out”) was connected to the knee extension model to actuate the knee torque. The knee angle output response was then fed back to the digital FFC for monitoring and tracking purposes.

### 3.4. Hardware Measurement Setup

**Figure 13:**
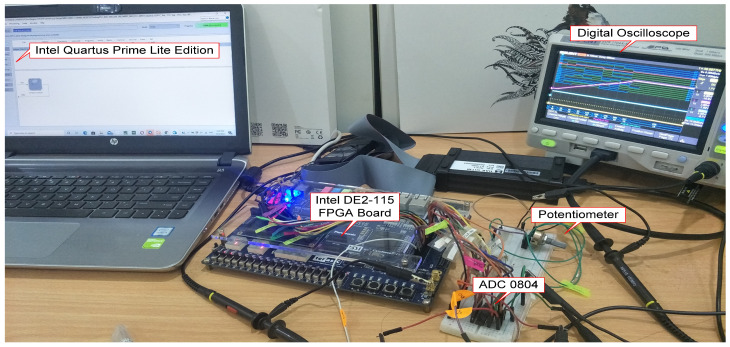
Hardware measurement setup using an Intel field-programmable gate array (FPGA) (DE2-115) board, an ADC (ADC0804) circuit, a rotary 10k ohm potentiometer and a digital oscilloscope.

Figure 13 depicts the hardware measurement setup for the designed FFC real-time verification purpose. The hardware setup mainly consists of an Intel FPGA (DE2-115) board, an ADC (ADC0804) circuit, a rotary 10k ohm potentiometer (Bourns, Inc., Reverside, CA, USA) and a digital oscilloscope (SIGLENT Technologies, Shenzhen, China). The designed FFC was first synthesized and downloaded to the FPGA (DE2-115) board. The ADC control output signals (“cs”, “wr”, “rd”), ADC 8-bit data and interrupt signals were connected from the FPGA board to the ADC (ADC0804) chip for the data acquisition process.

It should be noted that no actual human knee was used in this hardware measurement because the main goal was to test the functionality of the designed FFC in acquiring the feedback sensor data, processing the acquired data and producing a relevant pulse width signal based on the received feedback input. The resistive-based feedback sensor was replaced with a rotary 10k ohm potentiometer to represent the feedback sensor as well as knee angle movement.

Initially, the system was powered on and reset by pressing a reset button on the FPGA board. Then the potentiometer was rotated from 0 V to a maximum voltage of around 5.44 V. A single trigger measurement setting from the digital oscilloscope was used to acquire and track the changes in potentiometer voltage over time and capture the sequence of events of the measured signals on the digital oscilloscope screen. The changes in potentiometer voltage, as well as the ADC data over time, emulate the knee angle movement in the actual FES rehabilitation exercise. The defuzzy output (“defuzzy_out [7:0]”) and the generated pulse width modulation signal (“pwm_out”) were also measured to verify the designed digital FFC functionality in real-time.

## 4. Results and Discussions

This section discusses the results of synthesized digital FFC using the Intel FPGA (Cyclone IV E) chip (Terasic Inc., Hsinchu, Taiwan), register transfer level (RTL) simulation results in a digital simulator timing waveform, real-time hardware measurement, comparative analyses between hardware measurement and simulation results, and HDL co-simulations for system level verification in MATLAB Simulink. The results are explained sequentially in separate sub-sections.

### 4.1. Synthesized Digital Fuzzy Feedback Controller

**Figure 14:**
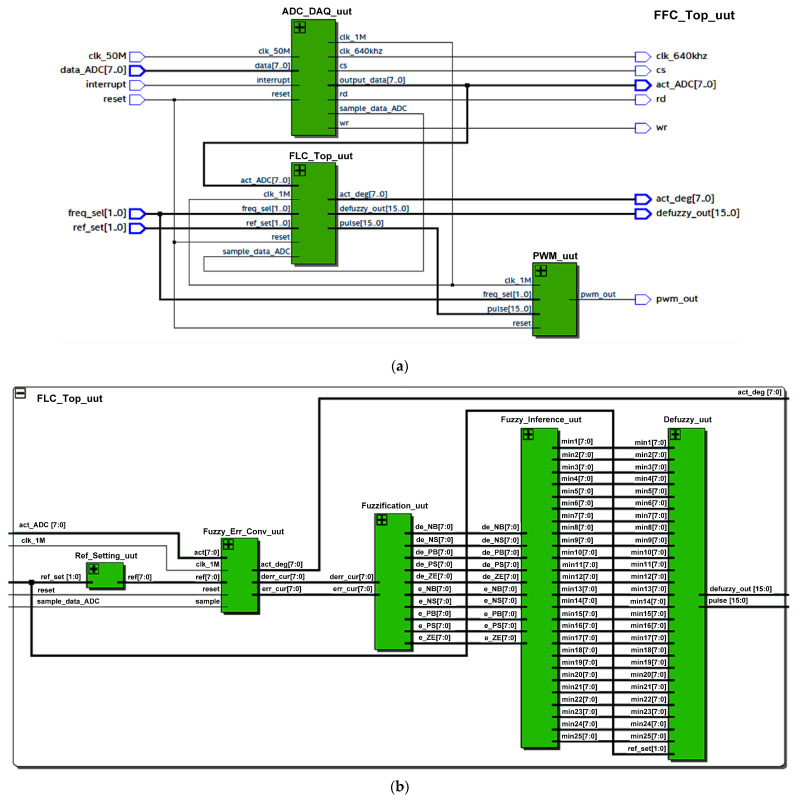
Schematics of the synthesized digital FFC using the Intel FPGA (Cyclone IV E) chip; (**a**) Internal architecture of the digital FFC; (**b**) Internal architecture of the digital FLC.

Figure 14 depicts the schematics of the synthesized digital FFC using Quartus II software (Intel, Santa Clara, CA, USA) for the Intel FPGA (Cyclone IV E) chip. The internal architecture of the synthesized digital FFC mainly consists of the ADC Data Acquisition (ADC_DAQ_uut), FLC (FLC_Top_uut) and PWM (PWM_uut) sub-modules as shown in Figure 14a. The internal architecture of the synthesized FLC is depicted in Figure 14b, which consists of Reference Settings (Ref_Setting_uut), Fuzzy Error Conversion (Fuzzy_Err_Conv_uut), Fuzzification (Fuzzification_uut) and Defuzzy (Defuzzy_uut) sub-modules.

Details on the FPGA family name, device types, logic elements utilization and maximum frequency (F_max_) that the design could operate are listed in Table 5. From the synthesized results obtained, it was observed that for this digital FFC design, the total number of logic elements used was 4544 (4% utilization), the total number of registers was 1593, the total number of pins was 52 (10% utilization), the total number of memory bits was 78,848 (2% utilization) and the total number of embedded multipliers was 19 (4% utilization). The maximum frequency (F_max_) that the system could operate at a low temperature (0 °C) was 103.08 MHz.

### 4.2. RTL Simulation of Digital Fuzzy Feedback Controller

**Figure 15:**
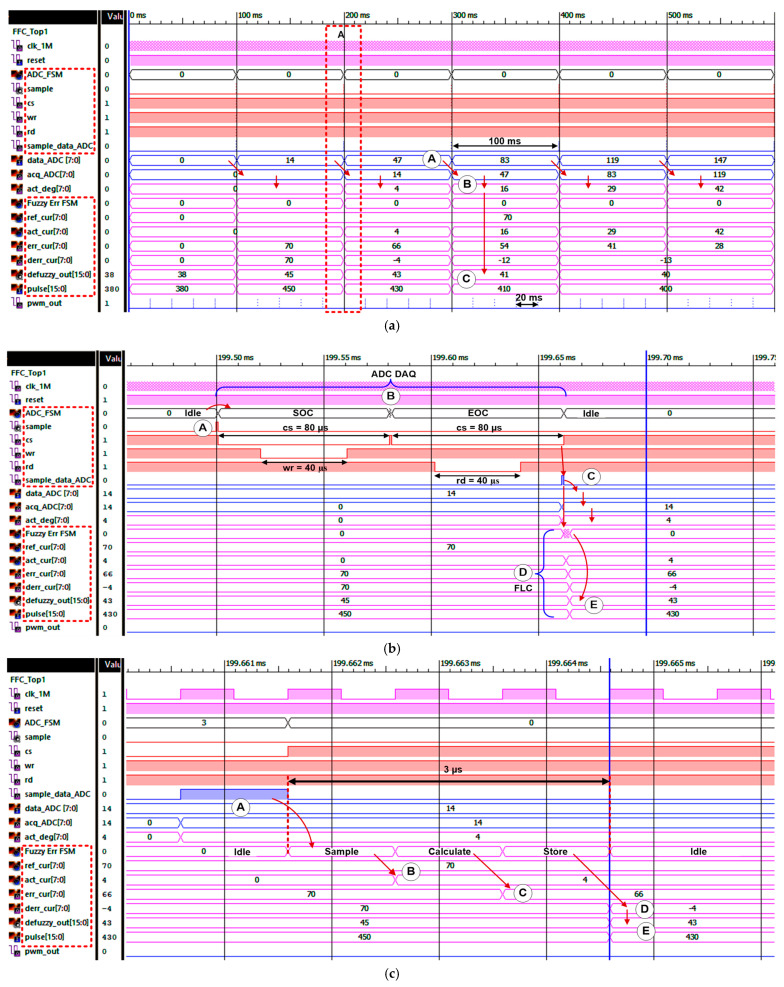
Register transfer level (RTL) simulation results of the digital FFC: (**a**) Overall FFC operation; (**b**) Zoom view into marked area A; (**c**) Zoom view into the FLC sub-module’s timing diagram.

The RTL simulation results were conducted using a digital timing wave viewer simulator to verify the designed digital FFC functionality. The ADC data acquisition and FLC operations are illustrated and analysed. Figure 15a depicts the overall digital FFC top level simulation results. As shown in the simulation result, every 100 ms new ADC data (“data_ADC [7:0]”) are acquired (refer to label A). The ADC data are then converted into degrees of angle (“act_deg [7:0]”) (refer to label B) before being processed by the FLC sub-module. The FLC sub-module then generates defuzzy output (“defuzzy_out [15:0]”) and pulse output (“pulse_out [15:0]”) every 100 ms according to the actual angle data acquired from the ADC (refer to label C). The stimulus pulse signal (“pwm_out”) is generated every 20 ms (stimulation frequency set at 50 Hz) based on the pulse output (“pulse_out [15:0]”) signals produced by the FLC sub-module.

The zoom view into marked area A is depicted in Figure 15b. As can be seen in Figure 15b, after the internal “sample” signal is activated (refer to label A), the FSM enters the SOC state and remains in this state for 80 µs for the ADC (ADC0804) chip to start the conversion process. Once the SOC state is completed, the FSM enters the Delay state only for a short duration (refer to label B) because the “interrupt” signal that emulates the ADC status signal is always set at logic LOW in the testbench. The FSM then moves to the EOC state for 80 µs for ADC data sampling purposes. Before the FSM moves from the EOC state to Idle state, the “sample_data_ADC” signal is activated (refer to label C). The external ADC data (“data_ADC [7:0]”) are then sampled and stored in the internal register (“acq_ADC [7:0]”) as indicated by the arrow. The “sample_data_ADC” signal also triggers the FLC sub-module to undertake the next operation (refer to label D). Based on the actual angle data (“act_deg [7:0]”), the FLC calculates the defuzzy output (“defuzzy_out [15:0]”) and pulse output ("pulse_out [15:0]”) signals (refer to label E) for 3 clock cycles only. The pulse output (“pulse_out [15:0]”) is then used by the PWM sub-module to generate the relevant pulse width signal (“pwm_out”) every 20 ms. Since the duration of the FLC sub-module’s operation is very short (3 clock cycles), the zoom view of the FLC timing diagram is shown in the next figure.

Figure 15c depicts the zoom view of the FLC sub-module’s timing diagram. Initially, when the “sample_data_ADC” is generated by the ADC FSM, the FLC FSM (Fuzzy Err FSM) moves from state Idle to state Sample (refer to label A). In the Sample state, the reference and actual angle data are stored in internal registers (“ref_cur [7:0]” and “act_cur [7:0]”) as shown in the timing waveform (refer to label B; “ref_cur = 70”, “act_cur = 4”). Then, the FSM moves to the Calculate state to calculate the Error (refer to label C; “err_cur = 70 − 4 = 66”). Thereafter, the FSM moves to the Store state, where the Change in Error is calculated (refer to label D; “derr_cur = 66 − 70 = −4”). Once completed, the FSM returns to the Idle state, where it will remain until the “sample_data_ADC” signal is activated again every 100 ms. The final defuzzy output (“defuzzy_out [15:0]”) and pulse output (“pulse_out [15:0]”) are calculated and produced concurrently by the FLC sub-module (refer to label E; “defuzzy_out = 43”, “pulse_out = 430”). From the timing diagram in Figure 15c, it is observed that the FLC sub-module requires only 3 µs to process the input angle data and produce the final defuzzy and pulse output data. This high-speed operation of the FLC sub-module guarantees the closed-loop FES system will be updated with a new pulse width value at the next stimulation cycle. Therefore, the pulse output data are always ready for the pulse width generation (“pwm_out”), which occurs every 20 ms for 50 Hz stimulation. The simulation results proved that digital FLC requires a very small timing operation (3 µs) compared to a microcontroller (218 µs) as has been published by Basith et al. (2017) [18].

### 4.3. Hardware Measurement and Comparative Analyses with Software Simulations

This section discusses the hardware measurement results of the ADC data acquisition process from the ADC (ADC0804) chip to the FPGA (DE2-115) board, the functionality of the designed digital FFC in tracking the feedback input data from the resistive feedback sensor (potentiometer), processing the digitized feedback data and producing an appropriate stimulus pulse width duration in real-time.

#### 4.3.1. ADC Data Acquisition

**Figure 16:**
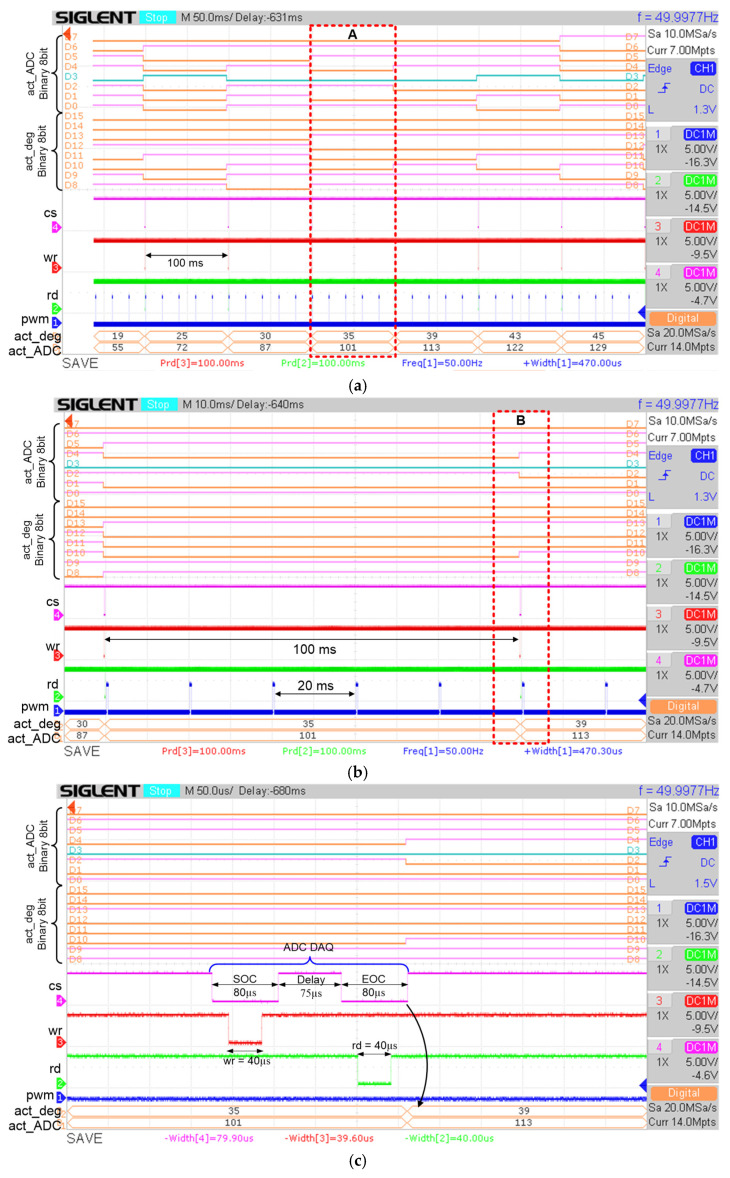
ADC data acquisition operation from the ADC (ADC0804) chip: (**a**) Overview of acquired ADC data every 100 ms; (**b**) Zoom view into marked area A; (**c**) Zoom view into marked area B.

Figure 16a depicts the overall data acquisition process from the ADC (ADC0804) chip every 100 ms. The output signals that are measured by the digital oscilloscope are control signals (“cs”, “wr”, “rd”) to communicate with the ADC (ADC0804) chip for the data acquisition process, the acquired 8-bit ADC data (“act_ADC”), the converted 8-bit ADC data to degree values (“act_deg”) and the PWM output signal (“pwm”).

The zoom view into marked area A is depicted in Figure 16b. The control signals (“cs”, “wr”, “rd”) are observed to be activated (logic LOW) every 100 ms to acquire the ADC data. As shown in Figure 16a,b, the ADC data (“act_ADC”) are acquired every 100 ms. The “pwm” signal is observed to be repeated every 20 ms, which is equivalent to 50 Hz. This is because the stimulus frequency is set to operate at 50 Hz. The zoom view into marked area B is depicted in Figure 16c. From the measured results, the total duration for the ADC data acquisition process is approximately 235 µs, which includes the SOC state (80 µs), Delay state (75 µs) and EOC state (80 µs) as shown in Figure 16c. Right after the EOC state, the new ADC data are acquired. This data acquisition process is repeated every 100 ms. Simultaneously, the acquired ADC data are converted into degree values as shown in Figure 16. For example, in Figure 16c, the ADC data with values of 101 and 113 are converted into degree values of 35° and 39°, respectively. The ADC to degree conversion is realized by using Equation (13) (Section 3.2.2).

#### 4.3.2. Hardware Measurements of Defuzzy and PWM Outputs

**Figure 17:**
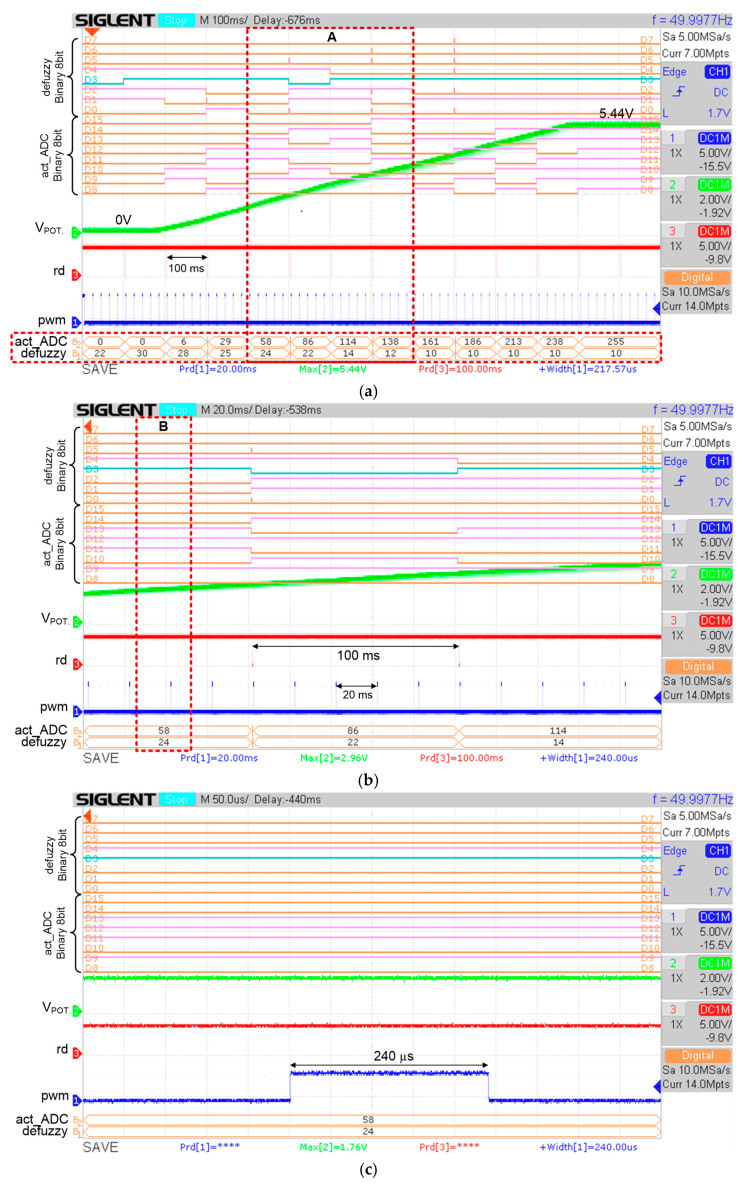
Hardware measurement of the digital FFC defuzzy output (“defuzzy”) from the acquired ADC data (“act_ADC”) every 100 ms. The actual knee angle movement is represented by the rotary 10k ohm potentiometer voltage (V_POT_). (**a**) Defuzzy output values for reference angle = 40°; (**b**) Zoom view into marked area A; (**c**) Zoom view into marked area B for a pulse width of 240 µs.

The functionality of the designed digital FFC in producing a correct defuzzy output and stimulus pulse width duration according to the acquired feedback input data were tested in real-time for all reference angels (70°, 40° and 30°). Figure 17 depicts an example of hardware measurement results for a reference angle with a value of 40°. The potentiometerwas rotated from a minimum voltage of 0 V to a maximum voltage of 5.44 V to emulate the knee angle movement from resting position (0°) to maximum angle (90°), respectively. In this measurement, a single trigger method was used to capture and track the sequence of events using the digital oscilloscope when the potentiometer (V_POT_) was rotated from 0 V to 5.44 V. As depicted in Figure 17a, the potentiometer voltage (V_POT_) starts at 0 V and increases to a maximum value of 5.44 V. During this event, the digitized potentiometer voltage (“act_ADC”) from the ADC (ADC0804) chip was acquired every 100 ms. The acquired ADC data (“act_ADC”), which ranges from 0 to 255, is equivalent to 0° to 90°, respectively. The defuzzy output (“defuzzy”) for each acquired ADC data were also captured on the digital oscilloscope screen to compare with the RTL and MATLAB simulation results. From the measured defuzzy output values (“defuzzy”) in Figure 17a, it is observed that as the ADC data increase, the defuzzy output values (“defuzzy”) vary according to the optimized fuzzy singleton MF output for the reference angle of 40° as listed in Table 3 (Section 3.1.3). The acquired ADC data represent the actual angle of knee movement.

Figure 17b illustrates the zoom view of marked area A from Figure 17a. One acquired ADC data are marked (refer to marked area B) to validate and correlate the generated PWM signal (“pwm”) with the defuzzy output (“defuzzy”). It is observed that the ADC data are acquired every 100 ms and the PWM signal is generated every 20 ms, which is equivalent to the stimulus frequency selection setting at 50 Hz.

Figure 17c depicts the zoom view of marked area B to further verify the duration of the generated PWM signal. It is observed that the duration of the PWM signal (“pwm”) is 240 µs which is equivalent to the defuzzy output (“defuzzy”) multiplied by 10 µs (24 × 10 µs) as mentioned in Section 3.2.6. As can be seen from Figure 17c, the defuzzy output is immediately produced after the ADC data are acquired. Based on the measured timing duration for ADC data acquisition (235 µs) obtained in Section 4.3.1 (Figure 16c) and the FLC processing time (3 µs) from Section 4.2 (Figure 15c), the total duration required for the digital FFC to produce the defuzzy output is approximately 238 µs.

#### 4.3.3. Comparative Analyses of Digital FLC Defuzzy Output between Hardware Measurement and Simulations

The defuzzy output data obtained from hardware measurements were compared with the RTL and MATLAB simulation results to check the accuracy of the designed digital FFC in processing the acquired ADC data and producing the defuzzy output values using integer data with a digital scaling format of 0 to 255. The real-time acquired ADC data from the hardware measurements (0 to 255) for three reference angles (70°, 40° and 30°) were saved and used as input data for the testbench in the RTL simulation.

Table 6 lists the FLC defuzzy output values obtained from real-time hardware measurement using the FPGA (DE2-115) board, RTL simulation and MATLAB (MAT) simulation for three reference angles (70°, 40° and 30°). Since the defuzzy output values obtained from the real-time FPGA measurement and RTL simulation are exactly similar for all reference angles, the defuzzy outputs for both are listed in the same column. The defuzzy output values obtained from MATLAB simulation and FPGA/RTL are almost the same with a very small difference in reading (less than 2), as shown in Table 6. The percentage of errors for each defuzzy output was calculated by using MATLAB simulation results as a reference. From the results shown in Table 6, it is observed that the maximum percentage of error is only 7%. The average percentage of errors between MATLAB (MAT) and FPGA/RTL for reference angles of 70°, 40°, and 30° is 1.3%, 1.0%, and 0.7%, respectively.

Since the RTL Simulation defuzzy output results are similar to the FPGA hardware measurement, it can be concluded that the designed digital FLC functions correctly when tested in real-time using the prototype FPGA board as a hardware measurement setup. Additionally, with the small average percentage of errors between MATLAB and real-time measurement (FPGA), it is proven that the designed digital FLC can produce an accurate pulse width duration to meet the reference target angle according to the specified fuzzy rules.

### 4.4. System Level Verification (HDL Co-Simulation)

**Figure 18:**
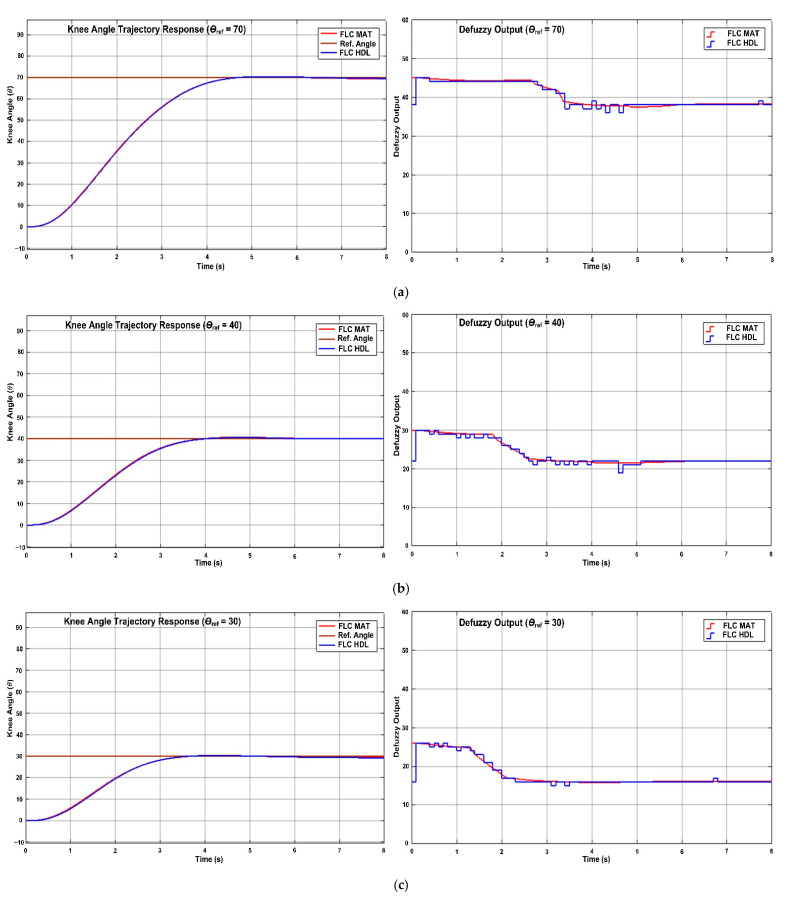
HDL co-simulation of knee angle trajectory and defuzzy output responses for three reference angles (*θ*_ref_): (**a**) Knee angle trajectory for *θ*_ref_ = 70°; (**b**) Knee angle trajectory for *θ*_ref_ = 40°; (**c**) Knee angle trajectory for *θ*_ref_ = 30°. Simulation response using MATLAB (FLC MAT), simulation response using HDL (FLC HDL) and reference angle (Ref. Angle).

Figure 18 depicts the HDL co-simulation results of the designed digital FLC (in HDL) and the modelled FLC (in MATLAB (Mathworks, Portola Valley, CA, USA)) using knee angle trajectory and defuzzy output responses for system level verification in the MATLAB Simulink environment. Both types of FLC controllers were employed in the closed-loop FES system with the knee extension model as a plant and tested at three different target angles (70°, 40° and 30°). Figure 18a depicts knee trajectory and defuzzy output responses for a target angle of 70°, Figure 18b depicts knee trajectory and defuzzy output responses for a target angle of 40° and Figure 18c depicts knee trajectory and defuzzy output responses for a target angle of 30°. From all the simulation results obtained (for three different target angles), it is observed that the modelled FLC and designed digital FLC are able to meet the target angle by using the optimized singleton MF settings. The knee trajectory and defuzzy output responses for the digital FLC are almost the same as for the modelled FLC, with an average percentage of error of 2.22% only.

The performance of the designed digital FLC for three different target angles was further investigated and compared with the modelled FLC by observing their knee trajectory response characteristics such as rise time, settling time, overshoot and steady-state error as listed in Table 7. For comparison purposes, the modelled FLC in MATLAB Simulink is abbreviated as FLC MAT, while the digital designed FLC in HDL is abbreviated as FLC HDL as shown in Table 7.

According to the trajectory response characteristics shown in Table 7, for smaller reference angles, the rise and settling times for both modelled FLC (FLC MAT) and digital FLC (FLC HDL) have become smaller. As the reference target angle increases, higher rise and settling times are required. The rise and settling times achieved for the digital FLC are almost the same as the modelled FLC for all reference angles. The rise time achieved is in the range of 2.00 s to 2.67 s and the settling time achieved is in the range of 3.26 s to 4.25 s for all reference angles (70°, 40° and 30°). The closed-loop FES system acquired a little bit longer settling time, especially at the reference angle of 70°, in order to achieve a better steady-state error. Both FLC MAT and FLC HDL have small and similar steady-state errors in the range of 0.3° to 0.4°. The low steady-state errors indicate better system performance. In terms of system overshoot, both FLC MAT and FLC HDL are able to maintain a very low overshoot (0.39° to 1.53°). The low overshoot represents a low distortion of the output signal.

Overall, the designed digital FLC in HDL has almost the same performance as the modelled FLC in MATLAB in terms of rise time, setting time, overshoot and steady-state error with very small differences. The average percentage of errors between FLC HDL and FLC MAT responses is less than 3% (2.22%). The rise and settling times are acceptable and reasonable according to the degree of reference angle settings. The overshoot and steady-state errors are small, which is very important to ensure the overall closed-loop FES system stability and better system performance in meeting the specified target reference angle. These performances show that the designed digital FLC in HDL is able to meet the performance of the simulated modelled FLC using MATLAB Simulink.

The performance of the designed digital FLC (FLC HDL) was further investigated and compared with other feedback controllers reported by other researchers, which include Benahmed et al. (2017) [59], Lynch and Popovic (2012) [1], Li et al. (2017) [25] and Watanabe et al. (2017) [28]. Table 8 shows the key performance characteristics of the knee trajectory response, such as rise time, settling time, overshoot and steady-state error.

From the trajectory response characteristics shown in Table 8, it is observed that for the reference angle of 40°, Benahmed et al. (2017) [59] and Lynch and Popovic (2012) [1] systems obtained low rise and settling times to achieve the desired target angle as compared to the FLC HDL, which requires 2.28 s for rise time and 3.70 s for settling time. However, the systems developed by Benahmed et al. (2017) [59] and Lynch and Popovic (2012) [1] have a higher overshoot and steady-state error when compared with the FLC HDL system, which has a 0.3° steady-state error and a 1.4° overshoot.

As for the reference angle of 30°, Li et al. (2017) [25] acquired a lower rise time (1 s) compared to the FLC HDL that required 2 s, but both systems required about the same settling time to achieve the desired target angle. However, the Li et al. (2017) [25] system settled with a little bit higher steady-state error (2°) compared to the FLC HDL system, which settled at a good steady-state error of 0.4° and an overshoot of 1.2°. For the reference angle of 20°, the Watanabe et al. (2017) [28] system performed with a relatively similar rise time and settling time but a higher steady-state error (0.6°) compared to the FLC HDL, which settled at 0.3° to 0.4° steady-state errors (for 40° and 30° reference angles).

In comparison to all other developed systems, both the developed modelled FLC (FLC MAT) and the digital FLC (FLC HDL) are able to achieve low steady-state errors (0.3° to 0.4°) and low system overshoot (1.2° to 1.4°). It is important to note that overshoot indicates the occurrence of the actual knee angle exceeding its target reference. This is due to the higher stimulus pulse width duration generated by the FLC. If the overshoot is bigger, this may lead to overstimulation, which consequently leads to early muscle fatigue. Larger steady-state errors for longer durations also indicate that the FLC provides an unnecessarily higher pulse width value. That higher pulse width value for a longer duration might also lead to overstimulation and early muscle fatigue. The designed digital FLC performance has shown small overshoot and steady-state errors, which ensure stability and no overstimulation of the closed-loop FES system. Additionally, a low steady error by the digital FLC indicates a good feedback control system, since the controller can provide an accurate stimulus charge to reach the target angle. For the closed-loop FES knee extension exercise, the most important criteria are that the patient can maintain the knee position at the desired target angle (set by the physician) for a certain duration and can perform the exercise repeatedly for a longer period of time. Therefore, low steady-state errors and small overshoots are very important to avoid overstimulation and early fatigue. In contrast to the exoskeleton, which is a wearable skeleton machine powered by external energy, the FES device uses the biological energy of the muscle to generate movement [59]. Hence, the feedback controller must be precise enough to accurately produce only the required amount of charge by regulating the pulse width duration to achieve the desired target angle [1,22,24,25].

## 5. Conclusions

The design and implementation of a digital FFC using the Intel FPGA (DE2-115) board for closed-loop FES knee extension movement has been thoroughly expounded. The FFC was designed to track and monitor the knee angle movement, to process the acquired knee angle data and provide an accurate amount of stimulus pulse width duration to meet the target reference angle. In this work, the FFC was designed to operate at three reference angles: 70°, 40° and 30°. Initially, the FLC was first modelled and tested using a non-ideal knee extension model as a plant in MATLAB Simulink. The initial modelling of FLC was to obtain optimum settings for membership functions and fuzzy rule sets. Then the optimized FLC settings were converted into a digital scale format for digital hardware implementation using HDL codes. The detailed internal design architecture of FFC, ADC data acquisition protocol, fuzzy error conversion protocol, implementation of FLC mathematical operation in hardware logic and overall design implementation flow have been illustrated and elucidated. The performance of the designed digital FLC was verified and compared with the modelled FLC using HDL co-simulation in the MATLAB Simulink environment. Finally, once the digital FLC was found to have similar performance to the modelled FLC in MATLAB Simulink, the digital FFC was designed by integrating the FLC with the ADC Data Acquisition sub-modules for real-time verification using an FPGA board.

The implementation of the designed digital FFC in the Intel FPGA (Cyclone IV E) chip utilized 4% of logic elements (4544/114,480 LEs), 2% of memory bits (78,848/3,981,312) and 4% of embedded multipliers (19/532). The maximum frequency of the designed FFC was 103.04 MHz at a low temperature of 0 °C. The computation time of the designed digital FLC was 3 µs as proven by the FSM operation (3 clock cycles) and RTL timing simulation results. From the hardware measurement results, the duration taken for the ADC data acquisition process was 235 µs. Therefore, the total processing time required by the digital FFC to produce the defuzzy output was approximately 238 µs. The high processing speed from the feedback controller (FLC) is very important to ensure the newly calculated pulse width duration can be updated immediately at the next stimulation cycle. The measured defuzzy output values from the FPGA-based digital FLC were also compared with the modelled FLC in MATLAB Simulink. The difference in values between the digital and modelled FLC was very small (less than 2), with a maximum average percentage of error of 1.3%. From the hardware measurement results, it can be concluded that the designed digital FFC can function correctly with high processing speed, precise timing control and high accuracy. The high processing speed with precise timing control is very important for the FES digital controller to realize parallel processing of the feedback data and flexible stimulus waveform generation to multiple electrodes synchronously, partially or asynchronously.

The system performance of the digital FLC was found to conform with the modelled FLC in terms of overall system response, rise time, settling time, overshoot and steady-state error with a very small difference between both systems. As the reference angle increases (from 30°, 40° to 70°), so do the rise and settling times. The digital FLC was found to have acceptable and reasonable rise and settling times according to the reference angle settings. Although the digital FLC has higher rise and settling times when compared to other research work, the digital FLC has a very small overshoot (1.2° to 1.4°) and settles into a very small steady-state error (0.3° to 0.4°). The performance characteristics of the designed digital FLC, with appropriate rise and settling times, little overshoot and very small steady-state error, are very important for a closed-loop FES system to ensure stability, avoid overstimulation of the stimulus charge and prevent early muscle fatigue.

Based on the encouraging findings obtained from simulations and hardware measurements, the designed digital FFC is feasible to be used for flexible multichannel closed-loop FES device development in neuromuscular applications. The designed FFC could be combined with the Digital Stimulator sub-module and the Biphasic Output Current Source circuit to develop a complete closed-loop FES device in the future.

## Figures and Tables

**Table 1:** Membership Function (MF) of Error (E) and Change in Error (dE) with digital scaling format (Decimal and Hexadecimal). The input MFs are Negative Big (NB), Negative Small (NS), Zero (ZE), Positive Small (PS) and Positive Big (PB).

MF	Error (E) and Change of Error (dE)	Digital Scaling	
		Decimal	Hexadecimal
NB	−20 to −10	0 to 63	$00 to $3F
NS	−20 to 0	0 to 127	$00 to $7F
ZE	−10 to 10	63 to 191	$3F to $BF
PS	0 to 20	127 to 255	$7F to $FF
PB	10 to 20	191 to 255	$BF to $FF

**Table 2:** Fuzzy rule-base table mapping for two inputs, Error (E) and Change in Error (dE) and singleton outputs. The input MFs are Negative Big (NB), Negative Small (NS), Zero (ZE), Positive Small (PS) and Positive Big (PB). The singleton output MFs are Very Small (VS), Small (SM), Medium (ME), Big (BG) and Very Big (VB).

		Input1—Error (E)				
**Input 2—Change in Error (dE)**		**NB**	**NS**	**ZE**	**PS**	**PB**
	**NB**	VS (min1)	VS (min2)	VS (min3)	SM (min4)	ME (min5)
	**NS**	VS (min6)	VS (min7)	SM (min8)	ME (min9)	BG (min10)
	**ZE**	VS (min11)	SM (min12)	ME (min13)	BG (min14)	VB (min15)
	**PS**	SM (min16)	ME (min17)	BG (min18)	VB (min19)	VB (min20)
	**PB**	ME (min21)	BG (min22)	VB (min23)	VB (min24)	VB (min25)

**Table 3:** Fuzzy output for reference angles of 70°, 40° and 30°. The singleton output MFs are Very Small (VS), Small (SM), Medium (ME), Big (BG) and Very Big (VB).

Singleton Position	Fuzzy Output [Ref = 70°]		Fuzzy Output [Ref = 40°]		Fuzzy Output [Ref = 30°]	
	Dec	Hex	Dec	Hex	Dec	Hex
VS	15	$0F	10	$0A	10	$0A
SM	20	$14	14	$0E	12	$0C
ME	38	$26	22	$16	16	$10
BG	42	$2A	24	$18	18	$12
VB	45	$2D	30	$1E	26	$1A

**Table 4:** ADC Data Acquisition state table.

Present States	Input	Next State	Output			
			cs	wr	rd	Sample_Data_ADC
Idle	Sample = 1	SOC	1	1	1	0
SOC	cnt = 80	Delay	0	0	1	0
Delay	Interrupt = 0	EOC	1	1	1	0
EOC	cnt = 80	Idle	0	1	0	1

**Table 5:** Synthesis summary report of the digital Fuzzy Feedback Controller (FFC) using the Intel field-programmable gate array (FPGA) (Cyclone IV E) chip.

Items	Types and Utilization
Family name	Cyclone IV E
Device	EP4CE115F29C7
Total Logic Elements	4544/114,480 (4%)
Total Registers	1593
Total pins	52/529 (10%)
Total Memory bits	78,848/3,981,312 (2%)
Embedded Multiplier 9-bit elements	19/532 (4%)
Total PLLs	0/4 (4%)
F_max_ (Slow 1200 mV 0 °C)	103.08 MHz

**Table 6:** Defuzzy output values from hardware measurement using the FPGA (DE2-115) board, RTL simulations and modelled FLC simulations in MATLAB (MAT) for three reference angles (70°, 40° and 30°).

Time (s)	FLC Defuzzy Output (70°)			FLC Defuzzy Output (40°)			FLC Defuzzy Output (30°)		
	FPGA/ RTL	MAT	Error (%)	FPGA/ RTL	MAT	Error (%)	FPGA/ RTL	MAT	Error (%)
0	38	38	0.0	22	22	0.0	16	16	0.0
0.1	45	45	0.0	30	30	0.0	26	26	0.0
0.2	43	43.8	1.8	29	29.4	1.4	25	25.2	0.8
0.3	41	41.2	0.5	24	24	0.0	17	17.8	4.5
0.4	40	40.8	2.0	24	24.6	2.4	16	16.2	1.2
0.5	40	40.8	2.0	22	22	0.0	12	12	0.0
0.6	41	41.9	2.1	14	14	0.0	10	10	0.0
0.7	30	31.6	5.1	12	12.9	7.0	10	10.3	2.9
0.8	18	19	5.3	10	10.3	2.9	10	10	0.0
0.9	15	15	0.0	10	10	0.0	10	10	0.0
1	15	15	0.0	10	10	0.0	10	10	0.0
1.1	15	15	0.0	10	10	0.0	10	10	0.0
1.2	15	15	0.0	10	10	0.0	10	10	0.0
1.3	15	15	0.0	10	10	0.0	10	10	0.0
	Avg Error (%)		1.3	Avg Error (%)		1.0	Avg Error (%)		0.7

**Table 7:** Performance of modelled FLC (FLC MAT) and digital FLC (FLC HDL) using the knee extension model in MATLAB Simulink.

Ref Angle	Rise Time		Settling Time (2%)		Overshoot (Deg)		Steady-State Error (Deg)	
	FLC MAT	FLC HDL	FLC MAT	FLC HDL	FLC MAT	FLC HDL	FLC MAT	FLC HDL
70	2.67	2.67	4.25	4.25	0.39°	0.46°	0.4°	0.4°
40	2.27	2.28	3.67	3.70	1.53°	1.40°	0.3°	0.3°
30	2.05	2.00	3.29	3.26	1.25°	1.19°	0.4°	0.4°

**Table 8:** Comparison of feedback controller performance from knee trajectory response.

Proposed by:	Type of Controller	Ref Angle (Deg)	Rise Time (s)	Settling Time (s)	Overshoot (Deg)	Steady State Error (Deg)
Benahmed et al. (2017) [59]	Adaptive Super Twisting	40°	0.61	0.90	11.4°	6.7°
Lynch and Popovic (2012) [1]	Sliding Mode	40°	0.46	1.19	12.6°	7.4°
Li et al. (2017) [25]	Adaptive Sliding Mode	30°	1.00	3.00	n/a	2.0°
Watanabe et al. (2017) [28]	Fuzzy Logic	20°	1.30	2.95	n/a	0.6°
FLC HDL (our work)	Fuzzy Logic	40°	2.28	3.70	1.4°	0.3°
FLC HDL (our work)	Fuzzy Logic	30°	2.00	3.26	1.2°	0.4°

## Data Availability

Not applicable.
